# Effects of Wood Ash on Nutrients and Heavy Metal(oid)s Mobility in an Ultisol

**DOI:** 10.3390/ijerph16071246

**Published:** 2019-04-08

**Authors:** Yeni Rodríguez, Bélgica Maudier, Erick Zagal, Pedro Hernández

**Affiliations:** 1 Departamento de Laboratorios de Investigacion y de Servicios, Corporación Colombiana de Investigación Agropecuaria (AGROSAVIA), Sede Central, Mosquera 250047, Cundinamarca, Colombia; yrodriguezg@agrosavia.co; 2 Faculty of Agronomy, University of Concepción, Vicente Méndez 595, Chillan 3780000, Chile; belgimaudier@udec.cl (B.M.); ezagal@udec.cl (E.Z.)

**Keywords:** cellulose waste, translocation, bioaccumulation, ryegrass, heavy metal mobility

## Abstract

Wood ash produced through cellulose manufacturing has agricultural uses due to its neutralizing power, like that of commercial products, in addition to providing key soil nutrients such as Ca, Mg, K, and P. However, this industrial waste can possess heavy metal(oid)s that bioaccumulate in the food chain. The objective of this study was to determine the effect of wood ash (WA) on the physicochemical properties of an Ultisol, the mobility of heavy metal(oid)s (As, Cd, Cr, Pb, and Ni) in the soil-plant-water system, and the nutritional response (N, P, and K) of ryegrass (*Lolium perenne* L.). The experiment was conducted in pots, under greenhouse conditions, using a completely randomized design. Ryegrass was grown in pots containing mixtures of WA plus soil at 7.5, 15, 30, and 75 g kg^−1^, commercial lime plus soil at 1.5 g kg^−1^, and unamended soil as a control. Heavy metal(oid)s were analyzed by inductively coupled plasma optical emission spectrometry (ICP-OES). All WA doses favored an increase in pH and the availability of P, Ca, Mg, Na, K, Cu, and Zn in soil and N, P, and K absorption in ryegrass. WA favored the availability and later absorption of heavy metal(oid)s by ryegrass (staying mainly in the roots). Heavy metal(oid)s mobility in the soil-plant-water system was as follows: Cr > Pb > Ni > As.

## 1. Introduction

The recovery of industrial wastes has increased worldwide due to their fertilizing properties, which are valuable to the agricultural industry, such as organic material content and elements crucial for plant nutrition [1]. This alternative also contributes to environmental well-being by decreasing the amount of waste sent to landfills, which are high-cost and hotspots for environmental contamination. Countries such as Finland, Portugal, Brazil, Canada, and Chile have led research to characterize and determine the potential agroforestry applications of alkaline wastes produced through cellulose manufacturing (e.g., wood ash, dregs, and grits), with a focus on the possible replacement of commercial alternatives, such as lime [2]. 

Wood ash produced through combustion is particularly promising as it increases soil pH and provides nutrients such as calcium (Ca), magnesium (Mg), potassium (K), and phosphorus (P) [1]. However, wood ash can also contain compounds detrimental to agricultural crops and the entire chain of production. Negative characteristics are directly dependent on the wood origin and the combustion technology used [1,3]. Among the possibly harmful components within ash are heavy metal(oid)s without any known biological function, including cadmium (Cd), lead (Pb), chromium (Cr), and arsenic (As). Similarly, trace elements, such as nickel (Ni), can be present, and, while these elements fulfill a biological role in small quantities, they can be toxic when present beyond optimal thresholds [4]. The agronomic relevance of these elements rests in the fact that they are non-biodegradable and persistent within the environment, causing bioaccumulation through the food chain and possible impacts to humans [5]. The prevalence of these elements in the soil depends on various factors, with some of the most important being soil pH, organic material content, texture, clay content, and the amounts of iron (Fe), aluminum (Al), and manganese (Mn) oxides [6].

Biological indicators, such as ryegrass, are used to evaluate the effects of applying ash as a liming material [7]. Plant species have different mechanisms for absorbing metal(oid)s through root tissue, either accumulating or translocating these elements depending on physiological and environmental factors [8]. Once inside the plant, metal(oid)s can affect physiological functioning, limiting nutrient absorption and decreasing crop yield.

Chile has no specific guiding or regulatory framework for the management or application of ashes in the agronomic sector. However, Chilean regulations such as Supreme Decree 148 [9] and Supreme Decree 4 [10] are used as references for the management of dangerous wastes and sewage sludge generated at wastewater treatment plants. Particularly, Supreme Decree 4 [10] establishes maximum heavy metal limits for organic sludge used in soils. These decrees have served to define the maximum application doses of waste-derived products in soils, thus protecting the environment and population health. 

Nevertheless, research is still needed to firmly define the optimal environmental dose for ash applications in soil. Such information will support the formulation of specific and local regulations for the use of cellulose industry wastes in the agroforestry sector. Toward these ends, this study evaluated the effects of wood ash on the physicochemical properties of an Ultisol under greenhouse conditions, assessing the yield and nutritional response of ryegrass, as well as the mobility of heavy metal(oid)s in the soil-plant-water system. 

## 2. Materials and Methods

### 2.1. Materials

Soil samples were collected at 20 cm depth from Arauco county, Chile (64° 71′ 80″ E; 58° 56′ 0.34″ S). The soil under study is derived from coastal marine sediments belonging to the Ultisol order, Merilupo series, classified as a mesic Humic Hapludults [11]. Wood ash (WA) samples were provided by the cellulose industry. The commercial lime (Cal Production Company of Chile, SOPROCAL) and ryegrass (*Lolium perenne* L.) seeds used as biological indicators were both obtained from the local market. 

### 2.2. Initial Characterization

Soil samples were dried at room temperature (<40 °C) and sifted to 2 mm [12]. The physicochemical properties of the soil were characterized as follows, according to the methodology of Sadzawka et al. [12]: The pH was at 1:2.5 (p/v) soil:water ratio; electrical conductivity (EC) at 1:5 (p/v) soil:water ratio; organic matter was determined by oxidation with dichromate (K_2_Cr_2_O_7_) and sulfuric acid (H_2_SO_4_); available nitrogen (N) by 2 M potassium chloride (KCl); available phosphorus (P) by the Modified Olsen Method; and aluminum (Al) saturation (%) was calculated through the formula Al/(∑ Ca, Mg, K, Na, Al) × 100, where Al was determined by 1 M KCl; exchangeable bases (Ca, Mg, K and Na) were determined by extraction with ammonium acetate pH 7, and available fraction of micronutrients (Fe, Mn, Zn, and Cu) (representing plant available forms of the elements) was assessed by extracting a portion of 10 g of soil with 20 mL of diethylenetriaminepentacetic acid (DTPA) extractant, adjusted to pH 7.3.

The WA was dried at 60 °C, macerated, and sifted to 0.5 mm [13]. Both WA and commercial lime were characterized as follows, according to the methodology of Zagal and Sadzawka [13]: The pH was at 1:5 (p/v); EC at 1:5 (p/v); organic matter was determined by oxidation with dichromate and sulfuric acid; and P, Ca, Mg, K, Na, Fe, Cu, Mn, and Zn were determined by calcination and acidic dilution. Neutralizing power (NP) was determined by titration with 1 M hydrochloric acid (HCl). 

The pseudo-total concentrations of metal(oid)s (As, Cd, Cr, Pb, and Ni) in the soil, lime, and WA were determined by microwave digestion with a 9:3 (v/v) mixture of concentrated ultrapure nitric acid (HNO_3_) and HCl (EPA Method 3051A) [14,15], using the Titan MPS Microwave Sample Preparation System (Perkin Elmer, Waltham, MA, USA). 

### 2.3. Experimental Design

A randomized complete block design was used with 18 experimental units of 1 kg soil (EU) consisting of the following 6 treatments, each with 3 replicates, as follows: WA7.5, WA15, WA30, and WA75 = wood ash at 7.5, 15, 30, and 75 g kg^−1^ respectively (equivalent to 15 t ha^−1^, 30 t ha^−1^, 60 t ha^−1^ and 150 t ha^−1^, respectively), Lime1.5 = commercial lime at 1.5 g kg^−1^ (equivalent to 3 t ha^−1^), and Control = unamended soil. The wood ash doses were selected according to preliminary unpublished data obtained under greenhouse conditions at the University of Concepción, Chile (these doses did not adversely affect the physicochemical properties of an Ultisol and radishes (*Raphanus sativus*) yield). The single lime dose (1.5 g kg^−1^) and 7.5 g kg^−1^ dose of WA were calculated to increase soil pH by one unit. Six treatments were evaluated for ryegrass (*Lolium perenne* L.).

All experimental work was performed in a greenhouse, located on El Nogal Experimental Station, belonging to the Faculty of Agronomy, University of Concepción (144 m.a.s.l., Ñuble Province, Chile).

### 2.4. Preparation of Amended Soil and Ryegrass Seeding Under Greenhouse Conditions

Homogenous mixtures of soil (1 kg) and amendments (WA at 7.5, 15, 30, and 75 g kg^−1^ and commercial lime at 1.5 g kg^−1^) were incubated in growth chambers (TS 606/3-I, WTW, Xylem Analytics, Germany), for 15 days at 22 °C, and moistened with distilled water until reaching between 60%–70% of field capacity (these groups are hereafter referred to as amended soil). Pots were filled with 1 kg of soil (amended soil and unamended soil) and three grams of ryegrass seeds (*Lolium perenne* L.) were sown (thus comprising the so-termed experimental unit (EU)). Soil moisture in the pots was maintained at 60%–70% by using a moisture sensor (N1500, Novus, Canoas, RS, Brazil) and distilled water (irrigation was applied manually). Once per week, excess water was applied to collect between 5–10 mL of percolating water, which was stored at 4 °C. Percolating waters from the entire irrigation process were mixed to obtain a single final sample, which was stored at 4 °C. At days 40, 70, and 100 post-seeding (between September 2015 and January 2016), cuttings were taken from the aerial shoot part of plant, approximately 5 cm above the soil surface. After the final cutting, the residual mass of each aerial shoot was collected. 

### 2.5. Final Characterization

#### 2.5.1. Analytical Preparation

Amended soil: After the experiment, the amended soil was air-dried (<40 °C) and sifted to 2 mm [12]. To determine the availability of heavy metal(oid)s (As, Cd, Cr, Pb, and Ni), the amended soil was macerated, sifted to 0.5 mm [13], and subjected to extraction with 0.1 M sodium nitrate (NaNO_3_) in a 1:10 (p/v) ratio and mechanical agitation for four hours [16]. The available fraction of micronutrients (Fe, Mn, Zn, and Cu) was assessed by extracting a portion of 10 g of amended soil with 20 ml of DTPA extractant, adjusted to pH 7.3.

Plant material (three cuttings + residual mass + roots): The plant material was washed with distilled water, dried with blotting paper, and weighed (wet weight). After this, the material was dried at 60 °C for three days and then weighed again (dry weight). These materials were ground and sifted to 1 mm (Mini Mill 5XBG00B, Thomas Wiley, Swedesboro, NJ, USA). Samples were stored in polyethylene flasks at room temperature until analysis. To determine the total metal(oid)s content (As, Cd, Cr, Pb, and Ni) an extraction was performed via digestion with concentrated ultrapure HNO_3_ and hydrogen peroxide (H_2_O_2_) in a 7:1 (v/v) ratio, according to the Microwave Digestion of Plants Method [15], using the Titan MPS Microwave Sample Preparation System (Perkin Elmer, Waltham, MA, USA).

Percolating water: To determine heavy metal(oid)s content (As, Cd, Cr, Pb, and Ni), the samples were filtered and acidified with HNO_3_ until they reached pH 2 [17].

#### 2.5.2. Measurements 

The physicochemical properties of amended and unamended soils were determined according to the methodologies presented in Section 2.2. Plant material (mixture of three cuttings + residual mass) was analyzed for N, P, and K contents, according to Sadzawka et al. [18], for total N according to the Kjeldahl Method and for total P and K by acidic calcination. The pH and EC of the percolating water were determined according to Sadzawka [17].

For all samples (i.e., amended soil, plant material, and percolating water), As, Cd, Cr, Pb, and Ni were quantified in triplicate using inductively coupled plasma optical emission spectrometry (ICP-OES) and the Optima 8000 model (Perkin Elmer, Waltham, MA, USA). Specifically, for the evaluation of As, samples were previously reduced from As^+5^ to As^+3^ using concentrated ultrapure 5% potassium iodide (KI, Merck), quantified using the hydride generation method [19]. A certified reference material was used for calibration purposes (WEPAL IPE 999, Wageningen Evaluating Programs for Analytical Laboratories of Wageningen University, Holland). Using this reference material and methodology, the recoveries of elements were 90%–100% and the following quantification limits were achieved: As, 0.72 µg L^−1^; Cd, 0.07 µg L^−1^; Cr, 1.08 µg L^−1^; Pb, 3.65 µg L^−1^; and Ni, 5.89 µg L^−1^.

### 2.6. Bioaccumulation Factor (BAF) and Translocation Factor (TF) of Heavy Metal(oid)s

At the end of the experiment, BAF and TF (both expressed as µg UE^−1^) [20] of heavy metal(oid)s in ryegrass were calculated according to the following formulae:

BAF = (Mp/Mi) × 100

TF = (Ma/Mr) × 100

where Mp is the total heavy metal(oid) in the plant (sum of plant material of cuttings at days 40, 70, and 100 post-seeding plus residual mass and roots on day 100 post-seeding); Mi is the initial total heavy metal(oid) in the soil and wood ash (sum of initial soil and wood ash); Ma is the total heavy metal(oid) in the aerial shoot part of the plant (sum of plant material of cuttings at days 40, 70, and 100 post-seeding plus residual mass on day 100 post-seeding); and Mr is the total heavy metal(oid) in the plant roots on day 100 post-seeding.

### 2.7. Statistical Analysis

Analysis of variance (ANOVA) was used to evaluate the results. The means of three replicates of each group were compared using the Tukey’s test with a 95% confidence level (none of the statistical analyzes incorporated the time variable (i.e., collection time of the samples, either for plant material or percolating water)). Linear regression analysis was used between metal(oid)s content in the plant material and soil pH as an effect of wood ash. All analyses were carried out in the Statistix 9.0 statistical package (Analytical Software, Tallahassee, FL, USA). 

## 3. Results and Discussion

### 3.1. Physicochemical Properties of the Soil and WA

The assessed soil presented characteristics typical of Ultisols (Table 1), including a moderately acidic pH (5.95), low Al saturation (16.44%), low P-Olsen, and low to mid-levels of exchangeable bases [21]. In contrast, the wood ash presented a basic pH (8.18); moderately high phosphorus; 65.79% of PN; high Ca, Mg, K, Na, Cu, and Zn contents; and very high Fe and Mn levels. Finally, the commercial lime presented a basic pH (12.5) and very high Ca content, which are common characteristics of liming agents.

Regarding the pseudo-totals of heavy metal(oid)s (i.e., As, Cd, Cr, Ni, and Pb) in the Ultisol, all were below the limits established by Supreme Decree 4 [10], except As (10 mg kg^−1^), which bordered on the established limit (Table 2). The presence of As in soils can result from anthropogenic activities, such as atmospheric deposition from industries and the indiscriminate use of fertilizers. Likewise, soil As content can naturally originate from a parent material [6]. On the other hand, the contents of heavy metal(oid)s in the commercial lime and wood ash samples were below the maximum limits established for organic amendments by Supreme Decree 4 [10].

### 3.2. Effect of WA on the Physicochemical Properties of Soil

After the 100 day experimental period, the physicochemical changes induced in the soil as a result of WA application were assessed (Table 3). When compared to the control, pH, EC, P, Ca, Mg, K, Na, Cu, and Zn significantly increased in the soil, in proportion to the WA dose, starting from 15 g kg^−1^. These results are in line with various previous reports [22,23,24]. The pH increase can be primarily attributed to WA contents of Ca and Mg, which were very likely present as oxides and carbonates, as well as to the particle size of WA (<0.5 mm), which would favor solubilization.

In turn, 7.5 g kg^−1^ WA and 1.5 g kg^−1^ commercial lime similarly impacted soil properties, causing analogous increases of pH, as compared to the control. A nearly neutral soil pH was obtained with the 30 g kg^−1^ WA, which is the value recommended for establishing ryegrass [25]. Finally, 75 g kg^−1^ WA caused a pH increase, which should be cautiously approached as this could cause P and micronutrient deficiencies due to precipitation or immobilization [26]. The increased EC at this dose could be directly related to greater Ca, Mg, and K contents from the WA which, in turn, could favor cation exchange capacity in soil [25].

Similar results have been reported by other authors. Over a one to five-year period, ash between 9 and 44 Mg ha^−1^ (equivalent to 4.5 to 22 g kg^−1^) and an application of 5% ash (equivalent to 50 g kg^−1^) increased pH by 2.1 units [1] and 1.9 units [2], respectively. Both studies postulated that increased pH resulted from the PN of wood ash, cation contributions, and reaction time. 

Regarding micronutrients, no significant changes in available Fe content were observed in the soil. However, Mn decreased (13%–21%) depending on the WA dose. Barman et al. [27] found similar behavior as an effect of lime application and postulated that this response is likely due to increased soil pH, which would result in less soluble, and consequently, less available, forms of Fe and Mn. As with micronutrients, organic matter did not significantly differ, as compared to the control. 

### 3.3. Effect of WA on the Physicochemical Properties of Percolating Water

**Figure 1:**
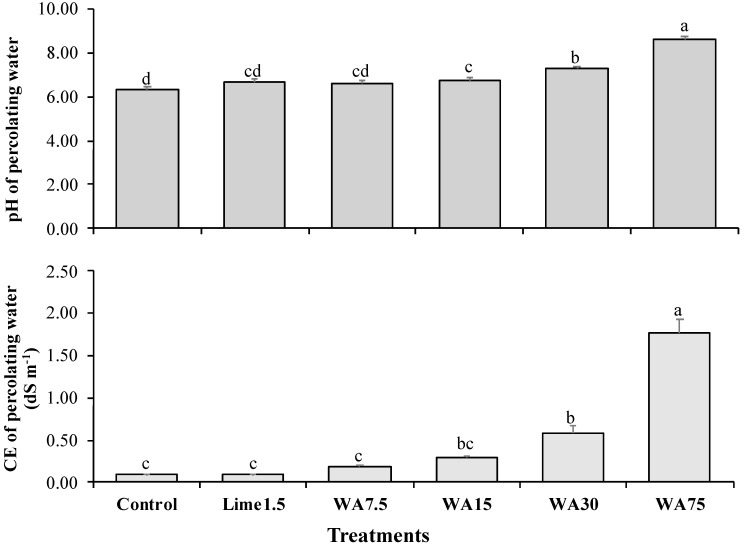
Effect of commercial lime and wood ash on pH and CE of percolating water. Lime1.5, commercial lime at 1.5 g kg^−1^; WA7.5, WA15, WA30 and WA75, wood ash at 7.5, 15, 30 and 75 g kg^−1^, respectively. Each bar represents the average of three repetitions, along with its standard error. Different letters (a, b, c, d) for each variable indicate significant differences, according to the Tukey test (*p* ≤ 0.05).

As seen in Figure 1, the pH and CE of percolating water increased directly proportional to the WA dose. The pH increased from 6.32 (control) to 8.63 (75 g kg^−1^ WA), showing significant differences from 15 g kg^−1^ WA. The pH 8.63 of percolating water obtained with 75 g kg^−1^ WA exceeded the maximum limit established by Supreme Decree 4 [10] (limit, pH 8.0). The CE increase from 0.09 dS m^−1^ (control) to 1.78 dS m^−1^ (75 g kg^−1^ WA) of percolating water did not exceed the limit for fresh water, established by the Geological and Mining Institute of Spain [28] (limit, EC 2.00 dS m^−1^). When comparing the effects of 1.5 g kg^−1^ commercial lime and 7.5 g kg^−1^ WA on the pH and CE of percolating water, no differences were observed.

### 3.4. Effect of WA on Ryegrass Yield and Nutritional Quality

**Figure 2:**
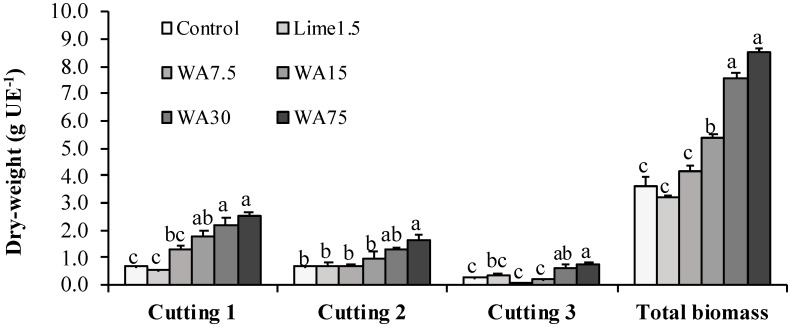
Dry weight of ryegrass (*Lolium perenne* L.). Cuttings 1, 2, and 3 were taken at days 40, 70, and 100 post-seeding, respectively. The total biomass includes the three cuttings plus the residual mass. Lime1.5, commercial lime at 1.5 g kg^−1^; WA7.5, WA15, WA30, and WA75, wood ash at 7.5, 15, 30, and 75 g kg^−1^, respectively. EU, experimental unit. Bars indicate standard error of the mean (n = 3). Means with different letters (a, b, c, d) differ significantly (Tukey’s test, *p* ≤ 0.05).

Ryegrass yield improved as an effect of WA treatments in the following order: The 1st cutting > 2nd cutting > 3rd cutting (Figure 2). These results can be attributed to a higher solubilization of WA nutrients during the first days of the experiment, concordant with greater nutritional demands by the ryegrass. The total biomass yield (three cuttings + residual mass) gradually increased from 3.64 to 8.49 g EU^−1^, following WA applications, which is significantly different from 15 g kg^−1^ WA. Dahlin et al. [29] confirmed that the use of wood ash improves biomass yield and affects nutrients availability in direct relation to the plant species, with positive effects for *Lolium perenne* L. and *Lolium multiflorum* L.

**Figure 3:**
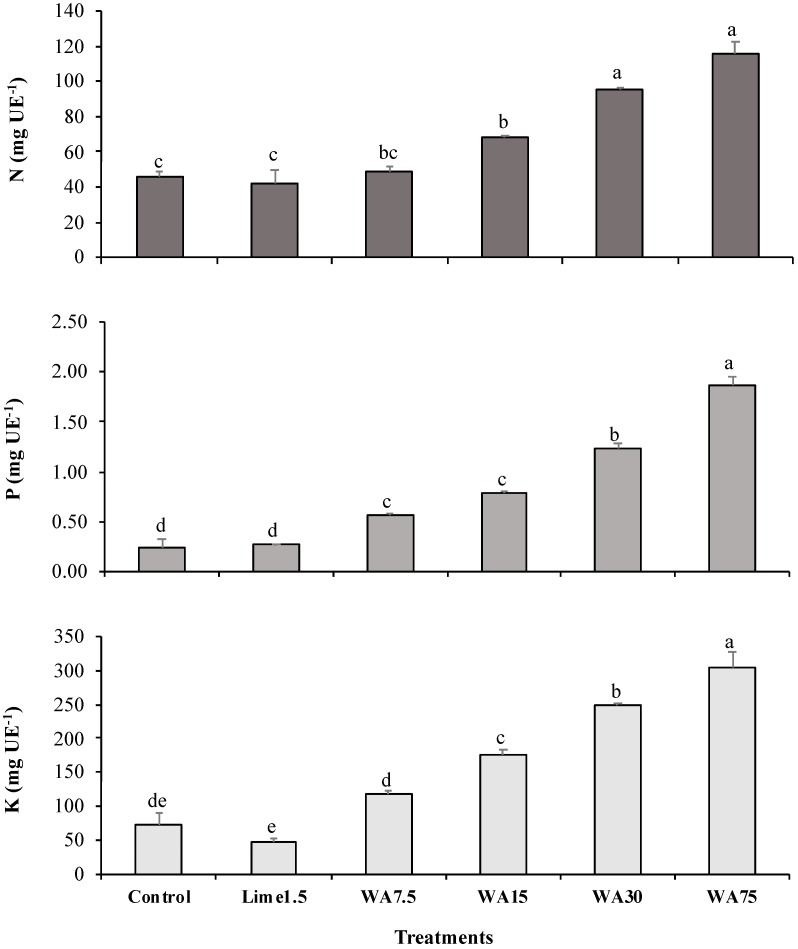
Contents of N, P, and K in ryegrass (*Lolium perenne* L.) following treatment with commercial lime and wood ash in Ultisol. Lime1.5, commercial lime at 1.5 g kg^−1^; WA7.5, WA15, WA30, and WA75: wood ash at 7.5, 15, 30, and 75 g kg^−1^, respectively. EU, experimental unit. Bars indicate standard error of the mean (*n* = 3). Means with different letters (a, b, c, d) differ significantly (Tukey’s test, *p* ≤ 0.05).

In the aerial shoot part of the plant, N, P, and K contents significantly increased following the application of WA (Figure 3). For N and K, significant differences were registered from the 15 g kg^−1^ WA, while P increased significantly from 7.5 g kg^−1^ WA.

In our results, the improved crop yield was dose-dependent of WA and can be related to increased N, P, and K, as well as to increased soil pH. Indeed, Arshad et al. [30] indicated that higher soil pH subsequently increased the solubilization of P provided through wood ash, all of which consequently provoked an improved barley yield (*Hordeum vulgare* L.).

Augusto et al. [1] proposed that increased soil pH would favor microbial activity and, thus, promote greater ammonification and nitrification, with consequent N availability. This situation was confirmed in the present study, starting from the 15 g kg^−1^ WA (Table 3, Figure 3). Particularly, these WA significantly increased soil pH and total N in the ryegrass. Considering that the results for N, P, and K in ryegrass were below sufficient levels, wood ash usage would need to be supplemented with nitrogenous or phosphatic fertilizers to make up for nutritional deficiencies [29].

### 3.5. Effect of WA on Heavy Metal(Oid)S Mobility in Soil and Percolating Water

The available forms of Cd, Cr, Ni, and Pb in the soil were lower than the quantification limits of the methodology (Table 4). Furthermore, dissolved forms of As, Cd, Cr, Ni, and Pb in the percolating water were also below the quantifiable limits (data not shown). These results indicate that a very low proportion of the metals provided by the soil and WA were mobilized by the liquid phase of soil and percolating water samples. According to the data recorded in Table 2 and Table 4, an estimated <1% of the heavy metal(oid)s were mobilized to liquid phases.

Violante and Caporale [31] postulated that when soil pH increases, the negative charges of the solid phase prevail, leading to the posterior absorption of divalent metals, an affinity that would be related to ionic potential. Furthermore, properties favoring the adsorption of cations and anions would include phyllosilicates contents, organic material levels, and the variable charges of minerals, such as Fe and Mn oxides. The consequent liberation of metal(oid)s in the medium would provoke competition for adsorption sites [31]. In the current study, it is possible that the very low mobilization of metal(oid)s could be associated with the strong adsorption favored by the charges of minerals inherent to Ultisol, as well as with the presence of Al, Fe, and Mn oxides.

Ochecova et al. [2] evaluated As, Cd, and Pb contents in a Cambisol treated with different fly ash doses (0–50 g ash per 5 kg soil, equivalent to 0–10 g kg^−1^). Their results showed no significant changes in the concentrations of available As, as occurred in this study. Cd and Pb decreased by 12.5% and 17%, respectively, as compared to the control, which contrasts with our results for Pb.

### 3.6. Metal(Oid)S Mobility in Ryegrass

#### 3.6.1. Metal(Oid)s Bioavailability and Absorption

Metal(oid)s concentrations in the root and aerial shoot parts of the plant were below the maximum consumption limits established by Spanish legislation for foraging animals (limits, As, 2 mg kg^−1^ and Pb, 10 mg kg^−1^) [32] (Table 4). Similarly, Cr levels were below the limits reported by Oliveira [33], who observed concentrations between 5 and 100 mg g^−1^ to be toxic for ryegrass. In turn, Cd contents were below the quantifiable limits of the methodology, a result that could be due to the low solubility of this metal at neutral and basic pH [34]. 

For As, Cr, Ni, and Pb (Table 4), the highest concentrations were mainly accumulated in ryegrass roots (>60% of absorbed metal(oid)s). With 30 and 75 g kg^−1^ WA, As and Pb contents were significantly different when compared to the control, whereas Cr and Ni were only significantly different with the 75 g kg^−1^ WA. As reported by Ogunkunle et al. [35], it has been suggested that root-focused accumulation of metals (e.g., Pb, Cu, Cr, Cd, and Zn) is a common phenomenon for grasses.

For As, Ni, and Pb contents in the aerial shoot part of ryegrass, no significant differences were found with respect to the control. However, Cr levels decreased with the 1.5 g kg^−1^ commercial lime and 7.5 g kg^−1^ WA. Violante and Caporale [31] proposed that such changes could be dependent on soil pH, where a near neutral pH would result in reduced absorption and a pH > 7 would increase the available Cr in the medium.

The accumulation of metal(oid)s in ryegrass was also analyzed through the BAF, which was <1% for all the evaluated heavy metal(oid)s (Table 5). This finding reflects a characteristic response of excluder plant species [20].

#### 3.6.2. Effect of WA on Heavy Metal(oid)s Translocation (TF)

TF for all heavy metal(oid)s was <100% (Table 5). Consequently, the physiological mechanism of ryegrass is indicative of an excluder plant [20]. This type of plant prevents the translocation of heavy metal(oid)s from the roots to the aerial shoot part of plant. Furthermore, ryegrass showed low arsenic translocation for all treatments. Zhao et al. [36] proposed that low As translocation could be related to the formation of complexes between As^3+^ and hydrogen sulfide (SH) groups present in root cells, which would finally be transported and stored in vacuoles.

TF for Cr decreased in the amended soils with respect to the control (from 42.5% to 16% on average). Likewise, TF for Ni and Pb decreased with WA treatments when compared to the control and commercial lime (Ni, from 52.5% to 23.4% and Pb, from 71.5% to 15.8%, all quantities on average). These results demonstrate that using WA as a liming agent facilitates a lower translocation of Cr, Ni, and Pb in ryegrass. Lou et al. [37] found that *Lolium perenne* L. present low metal translocation (4.4%–7.9%) when 3.2 mM Pb is applied to the soil. This indicates that the root acts as a primary barrier against translocation, possibly through precipitation to lead phosphate after transport into the cell interior.

### 3.7. Correlation Between Heavy Metal(Oid)S Mobility to Ryegrass and Soil pH

**Figure 4:**
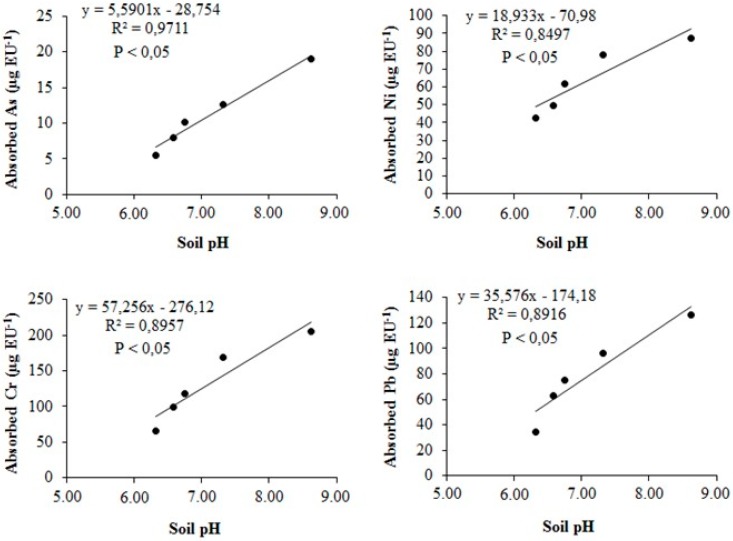
Linear relationship between the heavy metal(oid)s absorbed by ryegrass (*Lolium perenne* L.) and soil pH as an effect of wood ash treatments in the Ultisol. Absorbed metal is the sum of total metal in the root and aerial shoot parts of plant. Soil pH at 1:2.5 (w/v) corresponding to five treatments (Control and WA 7.5, WA15, WA30, and WA75 are shown in this same order in each graph).

The absorption of As, Cr, Ni, and Pb in the plants (i.e., three cuttings, residual mass, and roots) was positively correlated (R^2^ > 0.85) and with high significance (*p* < 0.05) to soil pH as a result of WA treatments (Figure 4). Effectively, Violante and Caporale [31] related metal(oid)s absorption with the oxidation state of each element, which is directly dependent on soil pH.

Changes in soil pH additionally affect the plant rhizosphere. Navarro-Aviñó et al. [5] reported that root exudates play an important role in metal(oid)s absorption. These organic compounds, particularly those with a high molecular weight (e.g., mucilages), might form an external root covering (i.e., mucigel) that would favor the complexing of Cd, Cu, and Pb. Additionally, Zhao et al. [36] indicated that ferrous oxides present in the soil could be oxidized by plant exudates, thereby forming iron hydroxide. This sheet could then bind to root tissue walls, where As in particular would present high affinity, resulting in accelerated absorption mechanisms.

## 4. Conclusions

WA favored heavy metal(oid)s availability and later absorption by ryegrass. The heavy metal(oid)s remained mainly in the roots and there was a very low translocation to the aerial shoot part of the plant. Heavy metal(oid) mobility in the soil-plant-water system was as follows: Cr > Pb > Ni > As, without exceeding the limits established for animals’ feeding. 

The optimal WA doses that did not negatively affect ryegrass growth in Ultisol were between 7.5 g kg^−1^ and 30 g kg^−1^. Seventy-five g kg^−1^ WA resulted in greater ryegrass yield, however pH levels in the soil and percolating water could be inadequate in the long-term because they could cause salinity and lowered nutrient availability in the soil. In turn, all WA doses favored increases in pH, P, Cu, Zn and exchangeable bases in Ultisol, which significantly increased absorption of N, P, and K in ryegrass.

Although the concentrations of the different metals found in the plant-soil-water system were low, it is important to note that a chronic exposure could cause toxic effects in plants and animals.

Future studies should consider secondary wood ash applications to soil in association with the stages of plant development. Furthermore, research is needed to assess the effects of wood ash doses in other soils types and other indicator plants with different bioaccumulation and translocation behavior.

## Figures and Tables

**Table 1:** Chemical properties of soil, wood ash, and commercial lime.

Parameter	Units	Ultisol	Wood Ash	Commercial Lime
pH	--	5.95 ^1^	8.18 ^2^	12.5 ^2^
EC	dS m^−1^	0.09 ^2^	8.47 ^2^	6.89 ^2^
OM	%	7.49	11.86	--
Al Saturation	%	16.44 ^3^	--	--
N	mg kg^−1^	22.60 ^3^	--	--
P	mg kg^−1^	4.00 ^3^	610.91 ^4^	--
K	cmol kg^−1^	0.21 ^3^	63.88 ^4^	--
Ca	cmol kg^−1^	2.27 ^3^	421.65 ^4^	1147.09 ^4^
Mg	cmol kg^−1^	0.83 ^3^	150.16 ^4^	40.54 ^4^
Na	cmol kg^−1^	0.04 ^3^	34.13 ^4^	--
Fe	mg kg^−1^	21.00 ^3^	22,200.00 ^4^	--
Mn	mg kg^−1^	33.80 ^3^	6,350.00 ^4^	--
Zn	mg kg^−1^	0.20 ^3^	48.00 ^4^	--
Cu	mg kg^−1^	0.70 ^3^	210.00 ^4^	--
NP ^5^	%	--	65.79 ^4^	91.80

Values are based on dry weights. ^1^ pH at 1:2.5 (w/v). ^2^ pH and EC at 1:5 (w/v). ^3^ Available elements in the soil. Organic matter (OM) was determined by oxidation with dichromate and sulfuric acid and quantified by colorimetry; Al saturation (%) = [Al/(∑ Ca, Mg, K, Na, Al) × 100]; Al was extracted with 1 M KCl; exchangeable bases (Ca, Mg, K, and Na) were extracted with ammonium acetate, pH 7, and micronutrients with diethylenetriaminepentacetic acid (DTPA), pH 7.3. Aluminum, interchangeable bases, and micronutrients were quantified by atomic absorption spectrophotometry. Available nitrogen = N-Nitrate + N-Ammonium, where N was extracted with 2 M potassium chloride (KCl) and quantified by colorimetry; P was determined by modified Olsen Method [12]. ^4^ Pseudo-total elements in wood ash and commercial lime were determined by calcination and acidic dilution and quantified by atomic absorption spectrophotometry [13]. ^5^ Neutralizing power (NP) was determined by titration with 1 M hydrochloric acid (HCl).

**Table 2:** Psuedo-total contents of heavy metal(oid)s in the Ultisol, wood ash, and commercial lime.

Heavy Metal(oid)s ^1^	Ultisol	Wood Ash	Commercial Lime	Max. Limit Allowed in Soils (pH > 5) ^2^	Max. Limit Allowed in Sewage Sludge ^2^
	mg kg^−1^				
**As**	10.00	5.00	0.90	10	40
**Cd**	0.30	0.90	0.17	2	40
**Cr**	38.20	25.45	0.95	NR	NR
**Pb**	12.00	13.00	7.68	50	400
**Ni**	13.10	15.30	2.29	30	420

^1^ Pseudo-total heavy metal(oid)s were extracted with a nitric acid and hydrochloric acid 9:3 (v/v) mixture and quantified by inductively coupled plasma optical emission spectrometry ICP-OES. ^2^ Maximum permissible limits of heavy metal(oid)s as established by Supreme Decree 4 [10]. NR, not regulated by Supreme Decree 4 [10].

**Table 3:** Changes in the chemical properties of soil as a result of wood ash application.

Parameter	Treatments					
	Control	Lime1.5	WA7.5	WA15	WA30	WA75
pH	6.32 ± 0.14 d	6.66 ± 0.12 cd	6.58 ± 0.18 cd	6.75 ± 0.15 c	7.31 ± 0.02 b	8.63 ± 0.10 a
EC (dS m^−1^)	0.09 ± 0.01 c	0.09 ± 0.00 c	0.18 ± 0.02 c	0.29 ± 0.02 bc	0.58 ± 0.09 b	1.78 ± 0.15 a
OM (%)	7.21 ± 0.31 a	5.87 ± 0.55 a	6.31 ± 0.09 a	6.40 ± 0.05 a	6.47 ± 0.21 a	6.25 ± 0.20 a
P (mg kg^−1^)	3.01 ± 0.07 d	2.96 ± 0.16 d	4.40 ± 0.26 cd	5.22 ± 0.47 c	9.11 ± 0.36 b	21.83 ± 0.98 a
K (cmol kg^−1^)	0.09 ± 0.00 d	0.11 ± 0.02 d	0.18 ± 0.01 cd	0.36 ± 0.02 c	0.78 ± 0.00 b	1.81 ± 0.11 a
Ca (cmol kg^−1^)	1.70 ± 0.27 d	3.96 ± 0.16 d	4.86 ± 0.15 cd	7.78 ± 0.14 c	11.19 ± 1.56 b	24.26 ± 0.56 a
Mg (cmol kg^−1^)	0.67 ± 0.02 d	1.02 ± 0.33 cd	1.22 ± 0.05 cd	1.58 ± 0.04 bc	1.96 ± 0.02 b	2.70 ± 0.08 a
Na (cmol kg^−1^)	0.04 ± 0.00 d	0.05 ± 0.01 d	0.08 ± 0.00 cd	0.16 ± 0.01 c	0.34 ± 0.00 b	0.79 ± 0.05 a
Fe (mg kg^−1^)	15.92 ± 0.40 a	18.60 ± 1.18 a	17.24 ± 0.70 a	16.71 ± 0.41 a	15.96 ± 1.23 a	17.66 ± 0.38 a
Mn (mg kg^−1^)	48.84 ± 3.51 a	43.01 ± 1.72 bc	42.65 ± 2.31 bc	44.57 ± 1.95 ab	39.10 ± 3.00 c	38.70 ± 1.50 c
Zn (mg kg^−1^)	0.16 ± 0.01 e	0.22 ± 0.02 de	0.34 ± 0.02 cd	0.45 ± 0.03 c	0.66 ± 0.02 b	1.02 ± 0.07 a
Cu (mg kg^−1^)	0.59 ± 0.02 d	0.86 ± 0.15 cd	0.91 ± 0.03 cd	1.14 ± 0.03 bc	1.47 ± 0.03 ab	1.52 ± 0.02 a

Values are based on dry weights, pH at 1:2.5 (w/v); EC at 1:5 (w/v). P, K, Ca, Mg, Na, Fe, Mn, Zn and Cu are available elements in the soil. Organic matter (OM) was determined by oxidation with dichromate and sulfuric acid and quantified by colorimetry. Exchangeable bases (Ca, Mg, K, and Na) were extracted with ammonium acetate, pH 7, and micronutrients with diethylenetriaminepentacetic acid (DTPA), pH 7.3. Exchangeable bases and micronutrients were quantified by atomic absorption spectrophotometry. Available nitrogen = N-Nitrate + N-Ammonium, where N was extracted with 2 M potassium chloride (KCl) and quantified by colorimetry. P was determined by Modified Olsen Method [12]. Lime1.5, commercial lime at 1.5 g kg^−1^; WA7.5, WA15, WA30 and WA75, wood ash at 7.5, 15, 30, and 75 g kg^−1^, respectively. Measurements are shown as the average of 3 replicates ± standard error. * Distinct letters groups (a, b, c, d) in the same row indicate significant differences (Tukey’s test, *p* ≤ 0.05).

**Table 4:** Heavy metal(oid)s content in the soil-plant system.

Element	Matrix	Treatments					
		Control	Lime1.5	WA7.5	WA15	WA30	WA75
		µg UE^−1^					
**As**	Soil	2.79 ± 0.87 a	2.54 ± 0.56 a	3.98 ± 0.52 a	2.47 ± 0.53 a	3.43 ± 0.32 a	2.94 ± 0.28 a
	Roots	1.43 ± 0.16 c	1.49 ± 0.35 c	2.20 ± 0.46 bc	2.76 ± 0.51 bc	3.40 ± 0.39 ab	5.30 ± 0.20 a
	Aerial Shoots	0.38 ± 0.06 a	0.46 ± 0.10 a	0.44 ± 0.09 a	0.63 ± 0.16 a	0.80 ± 0.25 a	1.05 ± 0.27 a
**Cr**	Soil	<QL	<QL	<QL	<QL	<QL	<QL
	Roots	15.45 ± 2.65 b	21.8 ± 4.36 b	29.35 ± 5.54 ab	34.55 ± 3.64 ab	48.54 ± 7.51 ab	59.75 ± 7.35 a
	Aerial Shoots	6.41 ± 0.32 bc	4.02 ± 0.55 d	4.54 ± 0.38 d	4.72 ± 0.28 cd	7.66 ± 0.74 ab	8.76 ± 0.51 a
**Ni**	Soil	<QL	<QL	<QL	<QL	<QL	<QL
	Roots	9.26 ± 0.70 b	10.86 ± 1.21 ab	13.62 ± 1.98 ab	16.38 ± 1.74 ab	21.49 ± 4.71 ab	23.61 ± 2.27 a
	Aerial Shoots	4.89 ± 0.59 a	5.37 ± 0.90 a	2.85 ± 0.47 a	4.15 ± 0.47a	4.53 ± 0.35 a	5.53 ± 0.39 a
**Pb**	Soil	<QL	<QL	<QL	<QL	<QL	<QL
	Roots	6.87 ± 0.91 c	11.7 ± 3.52 bc	18.67 ± 3.34 bc	20.79 ± 0.92 abc	28.24 ± 6.36 ab	36.74 ± 5.12 a
	Aerial Shoots	4.56 ± 0.75 a	6.14 ± 1.45 a	2.29 ± 0.68 a	4.35 ± 1.04 a	3.95 ± 0.72 a	5.32 ± 0.86 a

Heavy metal(oid)s in the soil correspond to available forms obtained by extraction with 0.1 M NaNO_3_ in a 1:10 (w/v) soil:solution ratio. Heavy metal(oid)s in the root and aerial shoot parts of the plant correspond to total forms obtained by extraction with a 7:1 (p/v) ratio of HNO_3_ and H_2_O_2_, quantified by inductively coupled plasma optical emission spectrometry (ICP-OES). Lime1.5, commercial lime at 1.5 g kg^−1^; WA7.5, WA15, WA30, and WA75, wood ash at 7.5, 15, 30, and 75 g kg^−1^, respectively. <QL, concentrations less than the quantification limit. Measurements are shown as the average of 3 replicates ± standard error. Distinct letter groups (a, b, c, d) in the same row indicate significant differences (Tukey’s test, *p* ≤ 0.05).

**Table 5:** Bioaccumulation factor (BAF) and translocation factor (TF) for heavy metal(oid)s in ryegrass (*Lolium perenne* L.).

Treatments	BAF				TF			
	As	Cr	Ni	Pb	As	Cr	Ni	Pb
	%							
**Control**	0.02	0.05	0.08	0.08	26.6	42.5	52.7	70.2
**Lime1.5**	0.02	0.06	0.09	0.13	33.4	18.9	52.3	72.8
**WA7.5**	0.03	0.07	0.09	0.15	24.4	13.8	20.9	12.2
**WA15**	0.03	0.08	0.12	0.18	26.1	13.8	25.5	21.4
**WA30**	0.04	0.12	0.15	0.22	22.7	18.5	23.5	14.3
**WA75**	0.06	0.14	0.16	0.28	20.3	15.0	23.6	15.2

Lime1.5, commercial lime at 1.5 g kg^−1^; WA 7.5, WA15, WA30, and WA75, wood ash at 7.5, 15, 30, and 75 g kg^−1^, respectively. BAF = (Mp/Mi) × 100, where Mp is the total heavy metal(oid) in the plant (sum of plant cuttings at days 40, 70, and 100 post-seeding plus residual mass and roots on day 100 post-seeding); Mi is the initial total heavy metal(oid) in the soil and wood ash (sum of initial soil and wood ash). TF = (Ma/Mr) × 100, where Ma is the total heavy metal(oid) in the aerial shoot part of the plant (sum of plant cuttings at days 40, 70, and 100 post-seeding plus residual mass on day 100 post-seeding); and Mr is the total heavy metal(oid) in the plant roots on day 100 post-seeding.
